# HPV Prevalence and Genotype Distribution Among Infertile and Fertile Women of Turkish Nationality and Association with Cytology and Vaccination Status

**DOI:** 10.3390/biomedicines13123108

**Published:** 2025-12-17

**Authors:** Ayfer Bakır, Büşra Demir Çendek, Muhammed Furkan Kürkçü, Çağlar İzmirli, Murat Aral

**Affiliations:** 1 Department of Medical Microbiology, Etlik City Hospital, Ankara 06170, Türkiye; furkankurkcu@gmail.com (M.F.K.); izmirli93.12@hotmail.com (Ç.İ.); aralmurat@hotmail.com (M.A.); 2 Department of Obstetrics and Gynecology, Etlik City Hospital, Ankara 06170, Türkiye; dr.busra_demir@hotmail.com

**Keywords:** human papillomavirus, infertility, genotype, high-risk HPV, PCR, HPV vaccine

## Abstract

**Background/Objectives:** Human papillomavirus (HPV) is one of the most common sexually transmitted infections, yet its role in female infertility remains uncertain. This study aimed to compare HPV prevalence and genotype distribution between infertile and fertile women and to evaluate demographic and clinical factors, together with HPV vaccine coverage, in both groups. **Methods:** Cervical samples from 200 infertile and 200 fertile women aged 18–45 years were analyzed for 28 HPV genotypes using multiplex real-time polymerase chain reaction (PCR). **Results:** HPV DNA was detected in 13.5% (27/200) of infertile women and 18.0% (36/200) of fertile women (*p* = 0.272). The most frequent genotypes were HPV-82 (5/200, 2.5%) and HPV-16 (5/200, 2.5%) in infertile women, and HPV-45 (8/200, 4.0%) and HPV-16 (7/200, 3.5%) in fertile women. Single HPV infections were more common in infertile women (81.5%, 22/27) than in fertile women (63.9%, 23/36). HPV positivity was not associated with reproductive, clinical, or lifestyle factors, and age-stratified analyses revealed no statistically significant differences (all *p* > 0.05). Among women aged 30–45 years, atypical squamous cells of undetermined significance (ASC-US) cytology was identified in eight infertile women, all of whom were HPV-negative, whereas one of nine fertile women with ASC-US was HPV-positive. No low-grade squamous intraepithelial lesion (LSIL) cases were detected in the infertile group. The 9-valent HPV vaccine covered 56.2% (18/32) of genotypes detected in infertile women and 45.1% (23/51) of those detected in fertile women. **Conclusions:** In this study, no significant differences were observed between the groups in terms of HPV prevalence, genotype distribution, or cytology findings. These results suggest that HPV is not an independent risk factor for infertility and highlight the need for further studies focusing on genotype-specific patterns, viral persistence, and biological mechanisms that may influence reproductive outcomes.

## 1. Introduction

Infertility is a major global public health concern, affecting approximately 8–15% of couples of reproductive age [1]. It results from multiple factors that impair reproductive function in either women or men. These factors include congenital and hormonal disorders, lifestyle-related determinants, environmental exposures, and psychosocial conditions [2,3,4,5].

Female infertility may arise from a variety of causes, such as ovulatory dysfunction, tubal obstruction, endometriosis, or sexually transmitted infections (STIs) [6,7]. STIs are often asymptomatic and may remain undetected for extended periods, potentially leading to irreversible impairment of reproductive functions. In particular, viral STIs such as human herpesvirus, human immunodeficiency virus, and human papillomavirus (HPV) have been suggested to affect reproductive health; however, these associations have not yet been fully elucidated [8,9,10].

HPV belongs to the *Papillomaviridae* family and is a double-stranded DNA virus with nearly 200 identified genotypes, of which approximately 40 are capable of infecting the genital tract. HPV genotypes are broadly classified into low-risk (LR), probable high-risk (pHR), and high-risk (HR) groups. LR types typically cause benign lesions, whereas HR types are associated with various anogenital and oropharyngeal cancers, particularly cervical cancer [11,12,13,14,15]. Among them, HPV types 16 and 18 play a pivotal role in the etiology of cervical carcinoma [15].

HPV infections are mostly asymptomatic, and many individuals remain unaware of their infection status. Although HPV infection does not necessarily lead to cellular lesions or proliferative changes, its potential to silently impair reproductive functions remains uncertain. This uncertainty underscores the need for further investigations to clarify the role of HPV detection in infertility diagnostics and to determine whether special management strategies are warranted for HPV-positive patients undergoing assisted reproductive procedures [16,17].

It has been particularly suggested that HPV infection may induce sperm DNA damage and apoptosis, thereby negatively affecting sperm viability, motility, and morphology; in addition, it may increase anti-sperm antibody levels, leading to impaired semen quality [10,18]. Moreover, HPV has been reported to infect trophoblast cells, resulting in embryonic apoptosis and implantation failures [10,17,18]. Although most HPV infections are eliminated by the immune system, some may become chronic, causing inflammation, cellular damage, and disruption of tissue integrity [19,20]. Previous studies have demonstrated that HPV may exert adverse effects on fertility through cervical inflammation and cellular alterations [21,22,23]. However, recent reviews emphasize that the role of HPV in infertility is not limited to cervical involvement but may also include detrimental effects on sperm parameters, failures in in vitro fertilization, and adverse pregnancy outcomes (e.g., premature rupture of membranes or miscarriages) [10,17]. These multifaceted effects indicate that HPV may influence both female and male fertility through direct and indirect mechanisms [10,17,18].

The prevalence and genotype distribution of HPV infections vary across geographical regions and age groups [24]. Although multiple causes of female infertility have been identified, the direct impact of HPV on fertility remains unclear, and the number of published studies on this topic is limited. Existing reports often demonstrate regional variations [1,18,25,26]. Some studies have suggested that HPV prevalence may be higher among infertile women, particularly for high-risk HPV genotypes; however, the underlying mechanisms remain unclear and have been hypothesized to involve chronic cervical inflammation, local immune alterations, impaired sperm-oocyte interaction, and potential disruption of implantation processes [1,7,25,27]. However, research conducted in this patient group in Türkiye is scarce [27]. This highlights the significance of obtaining epidemiological data on HPV prevalence and genotype distribution among infertile women in our country.

Evaluating the distribution of HPV vaccine-covered genotypes in the infertile female population is of critical public health importance. Studies demonstrating that HPV vaccination has no adverse effect on fertility suggest that these vaccines play a strategic role not only in cancer prevention but also in the preservation of reproductive health. The present study aims to compare HPV prevalence and genotype distribution between infertile and fertile women, analyze associated factors, and present findings on vaccine coverage in a manner that contributes both to clinical practice and to public health strategies.

## 2. Materials and Methods

### 2.1. Study Design

This cross-sectional comparative study included women aged 18–45 years who attended the Department of Gynecology and Infertility at Ankara Etlik City Hospital between November 2024 and March 2025. The study group consisted of 200 women diagnosed with infertility, while the control group comprised 200 women with no history of infertility who had experienced at least one live birth and were age-matched to the infertile group. Primary infertility was defined as the inability to achieve a clinical pregnancy despite at least 12 months of regular unprotected intercourse in women who had never conceived, whereas secondary infertility referred to infertility in women with a previous history of at least one clinical pregnancy. Of the infertile women included in the study, 155 (77.5%) were diagnosed with primary infertility, and 45 (22.5%) with secondary infertility. To increase the sample size and statistical power, women with primary and secondary infertility were combined into a single infertile group for the statistical analyses.

Thus, age compatibility between the two groups was ensured. The exclusion criteria were defined as a history of gynecological malignancy or premalignant lesions, prior HPV vaccination, active sexually transmitted infections, immunosuppressive disease or therapy, a history of surgical intervention on reproductive organs within the past six months, and the presence of severe psychiatric or neurological disorders. Written informed consent was obtained from all participants. Considering the HPV prevalence reported in the literature, the Cochran formula was applied [28]. Based on the power analysis, a minimum of 200 participants per group was targeted to achieve 80% statistical power at a 5% significance level. This study was conducted following approval from the Non-Interventional Scientific Research Committee of the Scientific Research Evaluation and Ethics Board of Ankara Etlik City Hospital (approval number: AEŞH-BADEK-2024-1068, Date: 30 October 2024). The research was performed in accordance with the principles of the Declaration of Helsinki, and informed consent was obtained from all participants.

### 2.2. Sample Collection

Cervical samples were obtained during gynecological examination using a sterile speculum and collected with Dacron-tipped sterile swabs. The samples were transferred into Viral Nucleic Acid Transport (VNAT) tubes (Bioeksen R&D Technologies Inc., Istanbul, Türkiye; Cat. No.: BS-NA-513m/BS-NA-513). All samples were stored at +4 °C and delivered to the molecular microbiology laboratory within 24 h. All procedures were performed in accordance with the guidelines of the Centers for Disease Control and Prevention (CDC).

### 2.3. Molecular Analysis

Since cervical swab samples were collected in the VNAT™ transport medium optimized to directly release nucleic acids, a separate DNA extraction step was not required prior to analysis. HPV DNA detection and genotyping were performed using the HPV Genotyping qPCR Kit developed by Bioeksen R&D Technologies Inc. (Istanbul, Türkiye). The analytical sensitivity of the kit was 99.5% and the specificity was 99.48% (Catalog No.: BS-GHPV-L-25/BS-GHPV-L-100, Revision Date: 4 September 2024/Rev.01). Using this real-time PCR (qPCR)-based kit, 28 distinct HPV genotypes were qualitatively identified. Signal detection was achieved through genotype-specific primers and probes targeting the E1, E2, E6, E7, and L1 regions of the HPV genome. The HPV genotypes targeted by the kit were classified into three groups according to the International Agency for Research on Cancer (IARC). The HR group included HPV-16, 18, 31, 33, 35, 39, 45, 51, 52, 56, 58, 59, and 68. The pHR group consisted of HPV-26, 53, 66, 67, 69, 73, and 82, while the LR group comprised HPV-6, 11, 40, 42, 43, 44, 54, and 61. All analyses were performed in accordance with the manufacturer’s protocol using the Magnetic Induction Cycler (MIC) Real-Time PCR system (Bio Molecular Systems, BMS, Upper Coomera, QLD, Australia). Data were automatically processed with the integrated Sigmoida Software (V8.6 REV.56). For quality assurance, each run included a negative control (NTC), a positive control (PC), and an internal control (IC), all of which were commercially supplied as part of the HPV Genotyping qPCR Kit by the manufacturer (Bioeksen R&D Technologies Inc., Istanbul, Türkiye; Catalog No.: BS-GHPV-L-25/BS-GHPV-L-100). Samples crossing the fluorescence threshold and displaying a sigmoidal amplification curve were considered positive, whereas those without amplification curves were classified as negative.

### 2.4. Cytology Assessment

Cervical cytology was evaluated in both infertile and fertile groups among women aged 30 years and older, in accordance with national and international screening guidelines. The results were classified according to the Bethesda system as follows: atypical squamous cells of undetermined significance (ASC-US), low-grade squamous intraepithelial lesion (LSIL), high-grade squamous intraepithelial lesion (HSIL), and negative for intraepithelial lesion or malignancy (Normal/Benign).

### 2.5. Statistical Analysis

All statistical analyses were performed using IBM SPSS Statistics v27.0 (IBM Corp., Armonk, NY, USA). Descriptive statistics were presented as counts and percentages for categorical variables. For group comparisons, the Pearson chi-square test was applied for categorical variables; Fisher’s exact test was used for 2 × 2 tables when the expected cell count was <5; and the extended Fisher–Freeman–Halton exact test with Monte Carlo simulation was employed for contingency tables larger than 2 × 2.

For comparisons of HPV positivity, risk categories (HR, pHR, LR), genotype distribution, infection type (single/multiple), age groups, and cytology results (≥30 years) between infertile and fertile women, odds ratios (ORs) with 95% confidence intervals (CIs) were calculated. All calculations were conducted at the “unadjusted OR” level. To reduce bias in OR estimates in cells with zero observations, the Haldane–Anscombe correction (0.5) was applied. ORs were computed using logarithmic transformation, and 95% CIs were obtained using the Wald method.

Uncorrected *p* values refer to *p* values obtained before adjustment for multiple comparisons. For multiple comparisons, extended Fisher tests with Monte Carlo simulation were performed. To control the false discovery rate (FDR) arising from multiple testing, the Benjamini–Hochberg correction was applied. A *p*-value of <0.05 was considered statistically significant.

## 3. Results

### 3.1. HPV Prevalence

HPV DNA was detected in 13.5% of infertile women (27/200; 95% CI: 9.5–18.9). In the fertile group, the corresponding rate was 18.0% (36/200; 95% CI: 13.0–24.1) (*p* = 0.272). Among infertile women, nine (4.5%) were non-Turkish nationals, whereas all women in the fertile group were Turkish nationals.

### 3.2. HPV Infection Type, Genotype Distribution, and Risk Categories

Among the 27 HPV DNA-positive infertile women, 13 distinct genotypes were identified. All detected genotypes in this group belonged exclusively to the HR or pHR categories, and no LR HPV genotypes were observed. Within this group, 88.9% (24/27) carried HR-HPV genotypes, while 37.0% (10/27) carried pHR-HPV types. The majority of infections in infertile women were single-genotype infections, whereas multiple infections mainly consisted of combinations of HR and pHR genotypes. The most common genotypes in the overall infertile cohort were HPV-82 (2.5%; 5/200) and HPV-16 (2.5%; 5/200), followed by HPV-31 (2.0%; 4/200) and HPV-51 (2.0%; 4/200).

In the fertile group, 20 different genotypes were detected among 36 HPV DNA–positive women. HR-HPV genotypes were present in all positive cases (100%; 36/36), followed by LR-HPV (25.0%; 9/36) and pHR-HPV types (16.7%; 6/36). Notably, LR-HPV genotypes were detected exclusively in fertile women and were predominantly observed in multiple infections. The identified LR-HPV genotypes were HPV-40, HPV-42, HPV-44, HPV-54, and HPV-61. The most prevalent genotypes in the overall fertile cohort were HPV-45 (4.0%; 8/200), HPV-16 (3.5%; 7/200), and HPV-51 (2.5%; 5/200).

**Figure 1:**
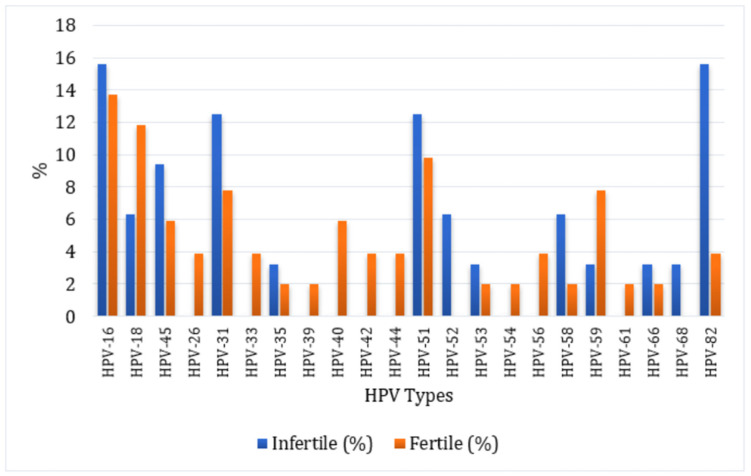
Genotype-specific distribution of HPV infection among infertile and fertile women.

Comprehensive comparisons of HPV positivity, genotype-specific distribution, infection type (single vs. multiple), and risk categories are summarized in Table 1, while Figure 1 illustrates genotype percentages based on total genotype detections in infertile (*n* = 32) and fertile (*n* = 51) women.

Percentages are presented based on the total genotype detections within each group (infertile: *n* = 32; fertile: *n* = 51). Of the 28 HPV genotypes tested, six genotypes (HPV-6, HPV-11, HPV-43, HPV-67, HPV-69, and HPV-73) were not detected in either group and therefore are not shown in the figure. No statistically significant differences were observed between infertile and fertile women for any HPV genotype (all *p* ≥ 0.05, Fisher’s exact test).

Regarding infection type, 81.5% (22/27) of HPV-positive infertile women had single infections and 18.5% (5/27) had multiple infections. The most common single genotype was HPV-16 (22.7%; 5/22), followed by HPV-51 (18.2%; 4/22) and HPV-31 and HPV-58 (9.1% each; 2/22). In multiple infections, HPV-82 (60.0%; 3/5) was the most frequent.

In fertile women, 61.1% (22/36) had single and 38.9% (14/36) had multiple infections. The most frequent single genotypes were HPV-16 (18.2%; 4/22) and HPV-51 (13.6%; 3/22), whereas HPV-18 was the most common in multiple infections, detected in 21.4% (3/14) of cases. A comparison of infection types (single vs. multiple) between infertile and fertile women is presented in Table 2.

### 3.3. Reproductive, Gynecological, and Lifestyle Characteristics of Infertile Women According to HPV Status

Table 3 summarizes reproductive, gynecological, and lifestyle characteristics exclusively among infertile women, as these variables are specific to infertility and not applicable to the fertile control group. Overall, no statistically significant associations were observed between HPV positivity and any of the evaluated characteristics. HPV status did not differ according to infertility type, body mass index (BMI), history of miscarriage, comorbidities, smoking status, alcohol consumption, or educational level (all *p* ≥ 0.05).

### 3.4. Distribution of HPV Infection Types Across Age Groups

In infertile women, the distribution of HPV DNA positivity varied across age groups (uncorrected *p* = 0.008), although this difference did not remain after FDR adjustment. Positivity was highest in the 19–25 years group (33.3%), with lower proportions in women aged 36–40 years (23.3%), 41–45 years (11.8%), 26–30 years (8.8%), and 31–35 years (6.6%). Fertile women showed no significant variation in HPV positivity across age categories (*p* = 0.098) (Supplementary Table S1).

When infection type was examined, infertile women showed an uneven distribution of single infections by age (uncorrected *p* = 0.021), but this pattern also lost significance after FDR correction. Multiple infections appeared only in the 19–25 and 36–40 years groups, though their distribution was not statistically meaningful. In the fertile group, neither single (*p* = 0.283) nor multiple infections differed across age groups (all *p* > 0.05).

At the genotype level, infertile women showed age-related clustering for HPV-31 (uncorrected *p* = 0.010; concentrated in women aged 36–40 years) and HPV-52 (uncorrected *p* = 0.021; detected only in the 19–25 years group). However, these patterns were not retained after FDR correction. No age-related differences were noted for other genotypes, including HPV-18 and HPV-82 (all *p* > 0.05). Among fertile women, genotype distributions were similar across all age groups (all *p* ≥ 0.05) (Supplementary Table S1).

### 3.5. Cytology Results and HPV Distribution

In women aged 30–45 years, no HPV positivity was detected in the ASC-US or LSIL categories among infertile women. In the fertile group, HPV DNA was identified in 11.1% (1/9) of ASC-US cases and in all women with LSIL (100%; 3/3). Among women with normal or benign cytology, HPV positivity was 11.6% (16/138) in the infertile group and 9.4% (10/107) in the fertile group, and this difference was not statistically significant (*p* = 0.677) (Supplementary Table S2).

### 3.6. Vaccine Coverage Rates by HPV Genotypes

Based on total genotype detections (infertile = 32, fertile = 51; overall = 83), the 9-valent HPV vaccine provided the broadest coverage: 56.2% (18/32) in infertile women, 45.1% (23/51) in fertile women, and 49.4% (41/83) overall. Coverage rates for the 2- and 4-valent vaccines were 21.9% (7/32) in the infertile group and 25.5% (13/51) in the fertile group, which reflected the absence of HPV-6/11 types in the study (Table 4). These coverage estimates are based on genotype detections rather than individual-level protection, as multiple HPV genotypes may be detected in a single woman.

Genotypes not included in current vaccines were detected in 43.8% (14/32) of infertile women, most commonly HPV-82 (15.6%; 5/32) and HPV-51 (12.5%; 4/32), followed by HPV-35, HPV-53, HPV-59, HPV-66, and HPV-68 (each 3.1%; 1/32). In fertile women, non-vaccine genotypes were observed in 54.9% (28/51), with the most frequent being HPV-51 (9.8%; 5/51) and HPV-59 (7.8%; 4/51). These were followed by HPV-40 (5.9%; 3/51) and HPV-26, HPV-42, HPV-44, HPV-56, and HPV-82 (each 3.9%; 2/51). Less common detections included HPV-35, HPV-39, HPV-53, HPV-54, HPV-61, and HPV-66 (2.0%; 1/51).

### 3.7. Odds Ratio (OR) Analysis of HPV Positivity and Infertility

When infertile and fertile women were compared in terms of overall HPV positivity, risk categories (HR, pHR, LR), HPV genotype distribution, infection type (single or multiple), age group-specific HPV distribution, and cytology results, no statistically significant differences were observed (Table 5).

## 4. Discussion

HPV infection is one of the most common sexually transmitted agents and is well recognized for its central role in cervical carcinogenesis [29]. In recent years, growing evidence has suggested that HPV may influence female reproductive health beyond its oncogenic potential [17,25,29]. In the present study, no significant differences were observed between infertile and fertile women in terms of HPV prevalence or genotype distribution. As one of the few studies evaluating HPV prevalence in infertile women in Türkiye, we found HPV DNA positivity in 13.5% of infertile women and 18.0% of fertile controls. These rates fall within the lower range reported in the meta-analysis by Yuan et al. [1].

The principal finding of this study is the absence of statistically significant differences between infertile and fertile women with respect to overall HPV positivity, risk categories, genotype profiles, infection patterns (single vs. multiple), and age-specific HPV distribution. These results suggest that HPV infection is unlikely to be an independent risk factor for infertility and align with findings from both national and international studies [1,22,30].

A substantial body of research has reported no meaningful association between HPV infection and female infertility. The comprehensive meta-analysis by Yuan et al. demonstrated no significant overall relationship between HPV infection and infertility when all genotypes were considered collectively (OR 2.13, 95% CI 0.97–4.65, *p* = 0.06) [1]. Similarly, a large Danish cohort study found no association between high-risk HPV infection and infertility [22]. Given the strong capacity of cohort studies to assess causality, these findings suggest that HPV is unlikely to act as a direct etiologic factor in female infertility. In line with this, an observational study involving women undergoing infertility treatment and oocyte donors reported no association between high-risk HPV positivity and pregnancy or miscarriage outcomes [30].

Data on Turkish women are limited; however, the findings from the few available studies generally support this conclusion. İleri et al. detected no significant difference in HPV positivity between infertile (11.5%) and control (12.1%) women [26]. Similarly, Ağar et al. reported that HPV prevalence did not differ significantly among women with unexplained infertility accompanied by chronic endometritis [31].

Nevertheless, several studies have suggested that the relationship between HPV infection and infertility may vary under specific conditions. Yuan et al. demonstrated that high-risk HPV infection, although not overall HPV infection, was significantly associated with infertility (OR 2.33, 95% CI 1.42–3.83, *p* = 0.0008) [1]. In addition, a large Taiwanese cohort reported an increased risk of infertility among HPV-positive women [6], while a case–control study from Iran identified a significantly higher HPV prevalence in infertile women (*p* = 0.005) and a markedly elevated infertility risk (OR = 5.30) [7]. In Türkiye, Okyay et al. observed a significant association between high-risk HPV infection and infertility among women with endometriosis [32], highlighting that underlying gynecological conditions may modulate the relationship between HPV infection and infertility.

Geographic and ethnic variability substantially influence HPV prevalence. For example, a study among infertile couples in China reported an HPV prevalence of 9.2%, with high-risk types accounting for more than 70% of infections [20]. In Brazil, reported HPV prevalence ranges widely from 13.7% to 54.3% across populations [33]. These observations demonstrate that HPV epidemiology varies by region and cultural context, underscoring the importance of considering geographic factors when interpreting infertility-related outcomes [34].

Differences among studies may also arise from varying definitions and durations of infertility, differences in sample size, and the distribution of infertility subtypes (primary, secondary, unexplained). In addition, diagnostic assays differ in sensitivity, specificity, and genotype coverage [1,6]. PCR-based methods typically provide higher sensitivity, whereas assays such as Hybrid Capture II may underestimate prevalence [35,36]. Del Prete et al. showed that switching from nested PCR to multiplex real-time PCR did not change overall HPV prevalence but did alter genotype distribution [37]. The exclusive use of cervical samples in our study may explain the relatively lower HPV positivity compared with studies that included broader anatomical sampling.

The nature of HPV infection is also important. Although single and multiple HPV infections were distinguished in the present study, the lack of detailed assessment of persistence limits the interpretation of HPV’s true impact. In addition, uncontrolled confounders such as sexual behavior, immune status, and smoking may further influence the results [1,6,29].

In this study, HPV positivity among infertile women did not show a consistent increase or decrease across age groups but was observed at relatively higher rates during certain age periods. This finding may be associated with variations in immune response, sexual behavior, or viral reactivation. Although the highest HPV prevalence was observed in the 19–25 age group, this difference did not remain statistically significant after FDR correction, possibly reflecting reduced statistical power due to multiple testing. Therefore, this apparent age-specific peak should be interpreted with caution. Nevertheless, the relatively higher HPV prevalence observed in younger women supports the importance of vaccination prior to the initiation of sexual activity.

HPV-82 is classified as a pHR genotype and has been reported less frequently in infertility-related studies. Although its clinical significance is not yet fully understood, emerging evidence suggests that non-vaccine pHR genotypes, such as HPV-82, may contribute to persistent infection and potentially influence reproductive outcomes, thereby warranting further investigation. In the present study, most HPV-positive infertile women presented with single infections, whereas a smaller proportion exhibited multiple infections. HPV-16 was the most frequent genotype in single infections, while HPV-82 predominated among multiple infections. Previous studies have suggested that the reproductive impact of HPV may vary by genotype and that multiple infections may influence fertility outcomes differently [1].

The relatively high detection rate of HPV-82 in infertile women is epidemiologically important, as this genotype is considered rare in most global HPV surveillance studies. Several factors may contribute to its elevated frequency in our cohort, including potential regional clustering, population-specific epidemiological patterns, and the high analytical sensitivity and broad genotype coverage of the multiplex real-time PCR assay used in this study. In addition, the relatively small number of HPV-positive infertile cases may have amplified the apparent proportion of less common genotypes. Although HPV-82 is not currently included in licensed vaccines, its frequent detection underscores the importance of continued genotype-based surveillance and suggests that regional genotype distributions should be considered when evaluating the long-term effectiveness of vaccination and screening strategies.

HPV-negative ASC-US cases were identified in the infertile group, consistent with prior research showing that abnormal cytology in infertile women is not always attributable to HPV infection [38,39]. Katki et al. reported that HPV-negative ASC-US carries a low risk of progression to advanced lesions [40]. These cases may represent non-HPV etiologies such as inflammation, other infections, or hormonal fluctuations [41,42,43]. While HPV testing is essential in ASC-US management, negative results should prompt evaluation for alternative causes.

HPV positivity was more common among infertile women with BMI ≥ 25 than in those with BMI < 25. Chronic inflammation and alterations in immune regulation may contribute to this difference, although findings in the literature remain inconsistent. Larger studies are needed to clarify this association [6,18,28,44,45].

Although HPV prevalence and genotype distribution did not differ between infertile and fertile women in our study, proposed mechanisms in the literature suggest that HPV may influence reproductive function. HPV has been hypothesized to impair oocyte quality, disrupt embryo development, and interfere with implantation [25]. Further elucidation of these biological mechanisms is essential for resolving discrepancies across studies.

This study has several limitations. The combination of primary and secondary infertility into a single analytical group represents a limitation of this study, as these conditions may differ in their underlying etiologies and clinical characteristics. This approach may have reduced the homogeneity of the infertile cohort and should be considered when interpreting the findings.

The single-center design and relatively small sample size limit the generalizability of the results. As the study was conducted in Ankara, the findings may not fully represent the broader Turkish population, given regional differences in HPV epidemiology. The cross-sectional design precludes causal inference, and reliance solely on cervical samples may have led to underdetection of infections in the upper genital tract. An additional limitation is the unequal distribution of nationality between the groups. A small proportion of the infertile group consisted of foreign women, whereas all participants in the fertile group were Turkish. The higher HPV positivity observed among foreign infertile women may have influenced overall prevalence estimates; therefore, differences in sexual behavior, healthcare access, and screening practices across nationalities should be considered as potential sources of bias. In addition, key HPV-related behavioral factors, such as number of sexual partners, age at sexual debut, and condom use, were not recorded, which may have resulted in residual confounding and should be considered when interpreting the findings.

Prospective, multicenter, large-scale studies are needed to clarify the potential etiological link between HPV infection and infertility. In addition, further molecular studies focusing on HPV persistence, viral load, and host immune responses are warranted to better elucidate the biological mechanisms underlying this association. Furthermore, large population-based studies indicate that HPV vaccination does not impair fertility [46]; therefore, vaccination should be encouraged for both individual-level and public health benefits.

## 5. Conclusions

In this study, HPV prevalence and genotype distribution were compared between infertile and fertile women, and no significant differences were detected between the groups. Overall HPV positivity, risk categories, infection patterns, genotype profiles, and age-specific distributions were similar in both groups, indicating that no evidence of an association between HPV infection and infertility was observed in this study population. Notably, the 9-valent HPV vaccine covered a substantial proportion of the genotypes detected in both groups; however, the frequent detection of non-vaccine genotypes such as HPV-82 highlights the importance of continued genotype-based surveillance. Future multicenter studies with larger sample sizes and longitudinal designs, as well as investigations focusing on HPV persistence, viral load, and host immune responses, are warranted to further elucidate the potential role of HPV in female infertility.

## Figures and Tables

**Table 1:** Distribution of HPV infection type, risk categories, and genotype-specific detections among infertile and fertile women.

	Infertile Women	Fertile Women	
Category	HPV-Positive Cases (*n* = 27)	HPV-Positive Cases (*n* = 36)	*p* Value
Infection type			
Single infection	22 (81.5)	22 (61.1)	>0.99
Multiple infection	5 (18.5)	14 (38.9)	0.057
Risk category			
HR-HPV (≥1)	24 (88.9)	36(100)	0.123
pHR-HPV (≥1)	10 (37.0)	6 (16.7)	0.445
LR-HPV (≥1)	0 (0.0)	9 (25.0)	0.004
pHR-HPV genotypes			
HPV-26	0 (0.0)	2 (5.6)	0.499
HPV-53	1 (3.7)	1 (2.8)	>0.99
HPV-66	1 (3.7)	1 (2.8)	>0.99
HPV-68	1 (3.7)	0 (0.0)	>0.99
HPV-82	5 (18.5)	2 (5.6)	0.449
HR-HPV genotypes			
HPV-16	5 (18.5)	7 (19.4)	0.771
HPV-18	2 (7.4)	6 (16.7)	>0.99
HPV-31	4 (14.8)	4 (11.1)	>0.99
HPV-33	0 (0.0)	2 (5.6)	>0.99
HPV-35	1 (3.7)	1 (2.8)	>0.99
HPV-39	0 (0.0)	1 (2.8)	>0.99
HPV-45	3 (11.1)	3 (8.3)	>0.99
HPV-51	4 (14.8)	5 (13.9)	>0.99
HPV-52	2 (7.4)	0 (0.0)	0.499
HPV-56	0 (0.0)	2 (5.6)	0.499
HPV-58	2 (7.4)	1 (2.8)	0.372
HPV-59	1 (3.7)	4 (11.1)	0.771
LR-HPV genotypes			
HPV-40	0 (0.0)	3 (8.3)	0.248
HPV-42	0 (0.0)	2 (5.6)	0.499
HPV-44	0 (0.0)	2 (5.6)	0.499
HPV-54	0 (0.0)	1 (2.8)	0.499
HPV-61	0 (0.0)	1 (2.8)	>0.99

Comparisons are shown for HPV-positive infertile (*n* = 27) and HPV-positive fertile (*n* = 36) women. Percentages are calculated based on the column denominator. Data are presented as *n* (%). Abbreviations: HPV = human papillomavirus; HR = high risk; pHR = probable high risk; LR = low risk. Statistical comparisons were performed using Pearson’s Chi-square test or Fisher’s exact test when appropriate. Statistical significance was set at *p* < 0.05.

**Table 2:** Distribution of HPV genotype combinations among infertile and fertile women.

Infection Type/ HPV Types	Risk Category	Infertile Women HPV+ Cases (*n* = 27), *n* (%)	Fertile Women HPV+ Cases (*n* = 36), *n* (%)	Total *n* (%)
Single infection		22 (81.5)	22 (63.6)	44 (30.2)
Multiple infection		5 (18.5)	14 (36.4)	19 (69.8)
HPV-16	HR	5 (18.5)	4 (11.1)	9 (2.2)
HPV-18	HR	1 (3.7)	2 (5.6)	3 (0.8)
HPV-26	pHR	0 (0.0)	1 (2.8)	1 (0.2)
HPV-31	HR	2 (7.4)	2 (5.6)	4 (1.0)
HPV-33	HR	0 (0.0)	2 (5.6)	2 (0.5)
HPV-35	HR	1 (3.7)	0 (0.0)	1 (0.2)
HPV-39	HR	0 (0.0)	1 (2.8)	1 (0.2)
HPV-42	LR	0 (0.0)	2 (5.6)	2 (0.5)
HPV-44	LR	0 (0.0)	2 (5.6)	2 (0.5)
HPV-45	HR	1 (3.7)	1 (2.8)	2 (0.5)
HPV-51	HR	4 (14.8)	3 (8.3)	9 (2.2)
HPV-53	pHR	1 (3.7)	0 (0.0)	1 (0.2)
HPV-58	HR	2 (7.4)	0 (0.0)	2 (0.5)
HPV-59	HR	1 (3.7)	0 (0.0)	6 (1.5)
HPV-66	pHR	1 (3.7)	1 (2.8)	2 (0.5)
HPV-68	pHR	1 (3.7)	0 (0.0)	1 (0.2)
HPV-82	pHR	2 (7.4)	1 (2.8)	3 (0.8)
HPV-16 + HPV-18	HR, HR	0 (0.0)	1 (2.8)	1 (0.2)
HPV-16 + HPV-59	HR, HR	0 (0.0)	1 (2.8)	1 (0.2)
HPV-16 + HPV-82	HR, pHR	0 (0.0)	1 (2.8)	1 (0.2)
HPV-18 + HPV-31	HR, HR	0 (0.0)	1 (2.8)	1 (0.2)
HPV-18 + HPV-51	HR, HR	0 (0.0)	1 (2.8)	2 (0.5)
HPV-18 + HPV-56	pHR, HR	0 (0.0)	1 (2.8)	1 (0.2)
HPV-18 + HPV-82	HR, pHR	1 (3.7)	0 (0.0)	1 (0.2)
HPV-26 + HPV-58	pHR, HR	0 (0.0)	1 (2.8)	1 (0.2)
HPV-31 + HPV-45	HR, HR	1 (3.7)	1 (2.8)	1 (0.2)
HPV-31 + HPV-52	HR, HR	0 (0.0)	0 (0.0)	1 (0.2)
HPV-35 + HPV-59	HR, HR	0 (0.0)	1 (2.8)	1 (0.2)
HPV-40 + HPV-54 + HPV-61	LR, LR, LR	0 (0.0)	1 (2.8)	1 (0.2)
HPV-40 + HPV-59	LR, HR	0 (0.0)	2 (5.6)	2 (0.5)
HPV-45 + HPV-51	HR, HR	0 (0.0)	1 (2.8)	1 (0.2)
HPV-45 + HPV-52	HR, HR	1 (3.7)	0 (0.0)	1 (0.2)
HPV-31 + HPV-82	HR, pHR	1 (3.7)	0 (0.0)	1 (0.2)
HPV-52 + HPV-82	HR, pHR	1 (3.7)	0 (0.0)	1 (0.2)
HPV-53 + HPV-56	pHR, HR	0 (0.0)	1 (2.8)	1 (0.2)

Abbreviations: HR = high risk; pHR = probable high risk; LR = low risk; HPV = human papillomavirus. Values are presented as *n* (%). Percentages in the infertile and fertile group columns were calculated among HPV-positive women only (infertile: *n* = 27; fertile: *n* = 36). Percentages in the single and multiple infection columns were calculated among HPV-positive women within each infection category. Percentages in the Total column are with respect to all women (*n* = 400).

**Table 3:** Reproductive, gynecological, and lifestyle characteristics at enrollment among infertile women stratified by HPV status.

Characteristic	HPV Positive *n* (%)	HPV Negative *n* (%)	*p* Value
BMI (kg/m^2^)			
<25	6 (8.0)	69 (92.0)	0.090
≥25	21 (16.8)	104 (83.2)	
Infertility type			
Primary	17 (11.6)	129 (88.4)	0.244
Secondary	10 (18.5)	44 (81.5)	
Abortion			
Yes	8 (22.2)	28 (77.8)	0.107
No	19 (11.6)	145 (88.4)	
Comorbidities			
Yes	1 (11.1)	8 (88.9)	>0.99
No	26 (13.6)	165 (86.4)	
Current smoking			
Yes	2 (10.0)	18 (90.0)	>0.99
No	25 (13.9)	155 (86.1)	
Alcohol use			
Yes	0 (0.0)	0 (0.0)	–
No	27 (13.5)	173 (86.5)	
Educational level, years			
<8	7 (12.5)	49 (87.5)	
8–10	14 (19.2)	59 (80.8)	0.164
>11	6 (8.5)	65 (91.5)	
Previous/current STI			
Yes	0 (0.0)	6 (100)	>0.99
No	27 (13.9)	167 (86.1)	

Abbreviations: BMI = body mass index; STI = sexually transmitted infection. Values are presented as *n* (%). Percentages were calculated within each row across HPV-positive and HPV-negative infertile women. Comparisons between groups were performed using the two-sided Fisher’s exact test for 2 × 2 tables or Pearson’s chi-square test for larger contingency tables. “–“ indicates not applicable. *p*-values are reported to three decimal places; values equal to 1.000 are shown as “>0.99”. Statistical significance was set at *p* < 0.05.

**Table 4:** HPV vaccine coverage based on genotype detections only.

Group	Vaccine	Covered GT Detections (*n*)	Coverage % (of Total Detections)
Infertile	2-valent	7	21.9
Infertile	4-valent	7	21.9
Infertile	9-valent	18	56.2
Fertile	2-valent	13	25.5
Fertile	4-valent	13	25.5
Fertile	9-valent	23	45.1
Total	2-valent	20	24.1
Total	4-valent	20	24.1
Total	9-valent	41	49.4

Notes: denominators—infertile women: total genotype detections = 32; fertile women: total genotype detections = 51; overall = 83. Percentages were calculated only from the total number of genotype detections, not from the number of women. Vaccine-included HPV genotypes: 2-valent (HPV-16, HPV-18); 4-valent (HPV-6, HPV-11, HPV-16, HPV-18); 9-valent (HPV-6, HPV-11, HPV-16, HPV-18, HPV-31, HPV-33, HPV-45, HPV-52, HPV-58). As HPV-6 and HPV-11 were not detected in the study population, the coverage rates of the 2-valent and 4-valent vaccines are identical. It should be noted that vaccine coverage rates were calculated based on genotype detections and do not represent individual-level protection, as multiple genotypes may be detected in a single individual.

**Table 5:** Odds ratio (OR) for the association between HPV positivity and infertility.

Category	Inf. HPV+	Inf. HPV−	Fer. HPV+	Fer. HPV−	OR	95% CI	*p* Value
HPV status (any HPV+)	27	173	36	164	0.71	0.41–1.22	0.272
Risk Category							
HR-HPV (≥1)	24	176	36	164	0.62	0.36–1.09	0.123
pHR-HPV (≥1)	10	190	6	194	1.70	0.61–4.77	0.445
LR-HPV (≥1)	0	200	9	191	0.00	0.003–0.87	0.004
HPV Types							
HPV-16	5	195	7	193	0.71	0.22–2.27	0.771
HPV-18	2	198	6	194	0.33	0.07–1.64	0.284
HPV-51	4	196	5	195	0.80	0.21–3.01	>0.99
HPV-31	4	196	4	196	1.00	0.25–4.06	>0.99
HPV-59	1	199	4	196	0.25	0.03–2.22	0.372
HPV-40 †	0	200	3	197	0.14	0.01–2.74	0.248
HPV-45	3	197	3	197	1.00	0.20–5.02	>0.99
HPV-26 †	0	200	2	198	0.20	0.01–4.15	0.499
HPV-33 †	0	200	2	198	0.20	0.01–4.15	0.499
HPV-42 †	0	200	2	198	0.20	0.01–4.15	0.499
HPV-44 †	0	200	2	198	0.20	0.01–4.15	0.499
HPV-56 †	0	200	2	198	0.20	0.01–4.15	0.499
HPV-82	5	195	2	198	2.54	0.49–13.24	0.449
HPV-35	1	199	1	199	1.00	0.06–16.10	>0.99
HPV-39 †	0	200	1	199	0.33	0.01–8.19	>0.99
HPV-54 †	0	200	1	199	0.33	0.01–8.19	>0.99
HPV-53	1	199	1	199	1.00	0.06–16.10	>0.99
HPV-58	2	198	1	199	2.01	0.18–22.35	>0.99
HPV-66	1	199	1	199	1.00	0.06–16.10	>0.99
HPV-61 †	0	200	1	199	0.00	0.013–8.19	>0.99
HPV-68 †	1	199	0	200	3.02	0.12–74.46	>0.99
HPV-52 †	2	199	0	200	3.02	0.12–74.46	>0.99
Infection type							
Single infection	22	178	22	178	1.00	0.53–1.87	>0.99
Multiple infection (≥2)	5	195	14	186	0.34	0.12–0.96	0.057
Age groups (years)							
18–25	8	16	13	37	1.42	0.49–4.10	0.586
26–30	6	62	11	32	0.28	0.10–0.83	0.028
31–35	4	57	6	34	0.40	0.10–1.51	0.188
36–40	7	23	3	27	2.74	0.63–11.82	0.299
41–45	2	15	3	34	1.51	0.23–10.00	0.645
Cytology							
ASCUS	0	8	1	8	0.33	0.01–9.40	>0.99
LSIL	0	0	3	0	NA	NA	NA
Normal/benign	16	122	10	97	1.27	0.55–2.93	0.677

Abbreviations: Inf = infertile; Fer = fertile; OR = odds ratio; CI = confidence interval; HR-HPV = high-risk HPV; pHR-HPV = probable high-risk HPV; LR-HPV = low-risk HPV; ASCUS = atypical squamous cells of undetermined significance; LSIL = low-grade squamous intraepithelial lesion. Notes: unadjusted odds ratios (ORs) with 95% confidence intervals (CIs) were calculated using Fisher’s exact test. The Haldane–Anscombe correction (†) was applied to zero cells. NA = not applicable, when no cases were present in one group. *p*-values are reported to three decimal places; values equal to 1.000 are presented as “>0.99”. Crude *p*-values are shown without multiplicity adjustment. Statistical significance was set at *p* < 0.05. Due to small or zero cell counts for several genotypes, some OR estimates may be unstable and should be interpreted with caution.

## Data Availability

Data supporting the findings of this study are available from the corresponding author, Ayfer Bakır, upon reasonable request.

## Supplementary Materials

The following supporting information can be downloaded at https://www.mdpi.com/article/10.3390/biomedicines13123108/s1, Supplementary Table S1: Age-stratified distribution of HPV status and genotypes among infertile and fertile women; Supplementary Table S2: Distribution of HPV infection and HPV types by cytology among infertile and fertile women aged ≥30 years.

## Abbreviations

ASC-US	Atypical squamous cells of undetermined significance
BMI	Body mass index
CI	Confidence interval
DNA	Deoxyribonucleic acid
Fer	Fertile
FDR	False discovery rate
HR-HPV	High-risk human papillomavirus
HPV	Human papillomavirus
IQR	Interquartile range
Inf	Infertile
LR-HPV	Low-risk human papillomavirus
LSIL	Low-grade squamous intraepithelial lesion
OR	Odds ratio
PCR	Polymerase chain reaction
pHR-HPV	Probable high-risk human papillomavirus
SD	Standard deviation
SPSS	Statistical Package for the Social Sciences
