# Supplementary material for: HPV Prevalence and Genotype Distribution Among Infertile and Fertile Women of Turkish Nationality and Association with Cytology and Vaccination Status
Source: Biomedicines. 2025 Dec 17;13(12):3108. doi: 10.3390/biomedicines13123108 (PMC12731213; doi:10.3390/biomedicines13123108)
Supplement: Supplementary file 1 [file biomedicines-13-03108-s001.zip › Supplementary Table S1.pdf]

Supplementary Table S1. Age-stratified distribution of HPV status and genotypes among infertile and fertile women

HPV Status / Type	Group	18–25, y (Inf=24, Fer=50)	26–30,y (Inf=68, Fer=43)	31–35,y (Inf=61, Fer=40)	36–40,y (Inf=30, Fer=30)	41–45, y (Inf=17, Fer=37)	Crude p-value	FDR p-value
HPV +	Inf.	8 (33.3)	6 (8.8)	4 (6.6)	7 (23.3)	2 (11.8)	0.008	0.185
HPV -	Inf.	16 (66.7)	62 (91.2)	57 (93.4)	23 (76.7)	15 (88.2)		
HPV +	Fer.	13 (26.0)	11 (25.6)	6 (15.0)	3 (10.0)	3 (8.1)	0.098	0.604
HPV -	Fer.	37 (74.0)	32 (74.4)	34 (85.0)	27 (90.0)	34 (91.9)		
Single	Inf.	6 (25.0)	5 (7.4)	4 (6.6)	5 (16.7)	2 (11.8)	0.021	0.194
Multiple	Inf.	2 (8.3)	1(1.5)	0 (0.0)	2 (6.7)	0 (0.0)		
Single	Fer.	6 (12.0)	7 (16.3)	5 (12.5)	2 (6.7)	2 (5.4)	0.283	0.703
Multiple	Fer.	7 (14.0)	4 (9.3)	1 (2.5)	1 (3.3)	1 (2.7)		
HPV-16	Inf.	0 (0.0)	1 (1.5)	3 (4.9)	1 (3.3)	0 (0.0)	0.617	0.913
HPV-16	Fer.	3 (6.0)	2 (4.7)	0 (0.0)	2 (6.7)	0 (0.0)	0.303	0.703
HPV-18	Inf.	1 (4.2)	1 (1.5)	0 (0.0)	0 (0.0)	0 (0.0)	0.603	0.913
HPV-18	Fer.	3 (6.0)	2 (4.7)	0 (0.0)	1 (3.3)	0 (0.0)	0.379	0.703
HPV-26	Inf.	0 (0.0)	0 (0.0)	0 (0.0)	0 (0.0)	0 (0.0)	-	0.913
HPV-26	Fer.	1 (2.0)	1 (2.3)	0 (0.0)	0 (0.0)	0 (0.0)	>0.99	0.738
HPV-31	Inf.	0 (0.0)	0 (0.0)	0 (0.0)	3 (10.0)	1 (5.9)	0.010	0.185
HPV-31	Fer.	1 (2.0)	1 (2.3)	0 (0.0)	0 (0.0)	2 (5.4)	0.475	>0.99
HPV-33	Inf.	0 (0.0)	0 (0.0)	0 (0.0)	0 (0.0)	0 (0.0)	-	0.185
HPV-33	Fer.	0 (0.0)	1 (2.3)	1 (2.5)	0 (0.0)	0 (0.0)	0.702	0.873
HPV-35	Inf.	1 (4.2)	0 (0.0)	0 (0.0)	0 (0.0)	0 (0.0)	0.207	0.615
HPV-35	Fer.	1 (2.0)	0 (0.0)	0 (0.0)	0 (0.0)	0 (0.0)	>0.99	0.999
HPV-39	Inf.	0 (0.0)	0 (0.0)	0 (0.0)	0 (0.0)	0 (0.0)	-	0.615
HPV-39	Fer.	0 (0.0)	1 (2.3)	0 (0.0)	0 (0.0)	0 (0.0)	0.747	>0.99
HPV-40	Inf.	0 (0.0)	0 (0.0)	0 (0.0)	0 (0.0)	0 (0.0)	-	-
HPV-40	Fer.	2 (4.0)	1 (2.3)	0 (0.0)	0 (0.0)	0 (0.0)	0.519	>0.99
HPV-42	Inf.	0 (0.0)	0 (0.0)	0 (0.0)	0 (0.0)	0 (0.0)	-	-
HPV-42	Fer.	0 (0.0)	2 (4.7)	0 (0.0)	0 (0.0)	0 (0.0)	0.141	0.615
HPV-44	Inf.	0 (0.0)	0 (0.0)	0 (0.0)	0 (0.0)	0 (0.0)	-	-
HPV-44	Fer.	0 (0.0)	0 (0.0)	2 (5.0)	0 (0.0)	0 (0.0)	0.097	0.615
HPV-45	Inf.	1 (4.2)	0 (0.0)	1 (1.6)	1 (3.3)	0 (0.0)	0.544	0.807
HPV-45	Fer.	0 (0.0)	0 (0.0)	2 (5.0)	0 (0.0)	1 (2.7)	0.216	0.615
HPV-51	Inf.	1 (4.2)	1 (1.5)	0 (0.0)	1 (3.3)	1 (5.9)	0.498	0.807
HPV-51	Fer.	1 (2.0)	2 (4.7)	1 (2.5)	0 (0.0)	1 (2.7)	0.883	0.913
HPV-52	Inf.	2 (8.3)	0 (0.0)	0 (0.0)	0 (0.0)	0 (0.0)	0.021	0.194
HPV-52	Fer.	0 (0.0)	0 (0.0)	0 (0.0)	0 (0.0)	0 (0.0)	-	-
HPV-53	Inf.	1 (4.2)	0 (0.0)	0 (0.0)	0 (0.0)	0 (0.0)	0.207	0.615
HPV-53	Fer.	1 (2.0)	0 (0.0)	0 (0.0)	0 (0.0)	0 (0.0)	>0.99	>0.99
HPV-54	Inf.	0 (0.0)	0 (0.0)	0 (0.0)	0 (0.0)	0 (0.0)	-	
HPV-54	Fer.	1 (2.0)	0 (0.0)	0 (0.0)	0 (0.0)	0 (0.0)	>0.99	>0.99
HPV-56	Inf.	0 (0.0)	0 (0.0)	0 (0.0)	0 (0.0)	0 (0.0)	-	-
HPV-56	Fer.	2 (4.0)	0 (0.0)	0 (0.0)	0 (0.0)	0 (0.0)	0.206	0.615
HPV-58	Inf.	0 (0.0)	1 (1.5)	0 (0.0)	1 (3.3)	0 (0.0)	0.798	0.913
HPV-58	Fer.	1 (2.0)	0 (0.0)	0 (0.0)	0 (0.0)	0 (0.0)	>0.99	>0.99
HPV-59	Inf.	0 (0.0)	0 (0.0)	0 (0.0)	1 (3.3)	0 (0.0)	0.356	0.807
HPV-59	Fer.	2 (4.0)	2 (4.7)	0 (0.0)	0 (0.0)	0 (0.0)	0.311	0.807
HPV-61	Inf.	0 (0.0)	0 (0.0)	0 (0.0)	0 (0.0)	0 (0.0)	-	-
HPV-61	Fer.	1 (2.0)	0 (0.0)	0 (0.0)	0 (0.0)	0 (0.0)	>0.99	>0.99

HPV-66	Inf.	1 (4.2)	0 (0.0)	0 (0.0)	0 (0.0)	0 (0.0)	0.207	0.615
HPV-66	Fer.	1 (2.0)	0 (0.0)	0 (0.0)	0 (0.0)	0 (0.0)	>0.99	>0.99
HPV-68	Inf.	0 (0.0)	1 (1.5)	0 (0.0)	0 (0.0)	0 (0.0)	>0.99	>0.99
HPV-68	Fer.	0 (0.0)	0 (0.0)	0 (0.0)	0 (0.0)	0 (0.0)	-	-
HPV-82	Inf.	2 (8.3)	2 (2.9)	0 (0.0)	1 (3.3)	0 (0.0)	0.210	0.615
HPV-82	Fer.	0 (0.0)	0 (0.0)	1 (2.5)	1 (3.3)	0 (0.0)	0.323	0.615

Abbreviations: Inf = infertile; Fer = fertile; y = years. Values are presented as number (%), calculated within each age group based on inf and fer. Comparisons were performed separately for infertile and fertile groups using the two-sided Monte Carlo Fisher's exact test. Statistical significance was set at $p<0.05$. For multiple comparisons, the Benjamini–Hochberg false discovery rate (FDR) correction was applied; values that were significant in the crude analysis (HPV positivity, HPV31, HPV52) did not remain significant after FDR adjustment. Values reported as "$p>0.99$" indicate that the test result was very close to 1.00 and no difference was observed between groups.
