# Supplementary material for: HPV Prevalence and Genotype Distribution Among Infertile and Fertile Women of Turkish Nationality and Association with Cytology and Vaccination Status
Source: Biomedicines. 2025 Dec 17;13(12):3108. doi: 10.3390/biomedicines13123108 (PMC12731213; doi:10.3390/biomedicines13123108)
Supplement: Supplementary file 1 [file biomedicines-13-03108-s001.zip › Supplementary Table S2.pdf]

Supplementary Table S2 Distribution of HPV infection and HPV types by cytology among infertile and fertile women aged ≥30 years

	**ASCUS (Inf, n=8)**	**ASCUS (Fer, n=9)**	**LSIL (Inf, n=0)**	**LSIL (Fer, n=3)**	**Normal/Benign Inf, n=138)**	**Normal/Benign (Fer, n=107)**
HPV positive	0 (0.0)	1 (11.1)	0 (0.0)	3 (100)	16 (11.6)	10 (9.4)
HPV negative	8 (100)	8 (88.9)	0 (0.0)	0 (0.0)	122 (88.4)	97 (90.6)
HPV types (counts)						
HPV-16	0	0	0	2	5	0
HPV-18	0	0	0	0	0	1
HPV-31	0	0	0	0	2	1
HPV-33	0	0	0	0	0	1
HPV-44	0	0	0	0	0	2
HPV-45	0	0	0	1	1	0
HPV-51	0	1	0	0	2	0
HPV-58	0	0	0	0	2	0
HPV-59	0	0	0	0	1	0
HPV-68	0	0	0	0	1	0
HPV-82	0	0	0	0	0	1
HPV-16+HPV-59	0	0	0	0	0	1
HPV-16+HPV-82	0	0	0	0	0	1
HPV-31+HPV-45	0	0	0	0	1	1
HPV-31+HPV-82	0	0	0	0	1	0
HPV-45+HPV-51	0	0	0	0	0	1

Abbreviations: Inf = infertile; Fer = fertile; ASC-US = atypical squamous cells of undetermined significance; LSIL = low-grade squamous intraepithelial lesion; HPV = human papillomavirus. Values are presented as counts or n (%). A dash (-) indicates not applicable due to zero cases in that cytology category. HPV type rows indicate the number of detections within each cytology category and group.
